# Approaches to health-care provider education and professional development in perinatal depression: a systematic review

**DOI:** 10.1186/s12884-017-1431-4

**Published:** 2017-07-24

**Authors:** Laura E. Legere, Katherine Wallace, Angela Bowen, Karen McQueen, Phyllis Montgomery, Marilyn Evans

**Affiliations:** 1 Registered Nurses’ Association of Ontario (RNAO), 158 Pearl Street, Toronto, ON M5H 1L3 Canada; 2 University of Saskatchewan, 104 Clinic Place, Saskatoon, SK S7N 2Z4 Canada; 3 Lakehead University, 955 Oliver Road, Thunder Bay, ON P7B 5E1 Canada; 4 Laurentian University, 935 Ramsey Lake Road, Sudbury, ON P3E 2C6 Canada; 5 University of Western Ontario, 1151 Richmond Street, London, ON N6A 3K7 Canada

**Keywords:** Pregnancy, Postpartum, Perinatal, Antenatal, Depression, Education, Health-care provider

## Abstract

**Background:**

Perinatal depression is the most common mental illness experienced by pregnant and postpartum women, yet it is often under-detected and under-treated. Some researchers suggest this may be partly influenced by a lack of education and professional development on perinatal depression among health-care providers, which can negatively affect care and contribute to stigmatization of women experiencing altered mood. Therefore, the aim of this systematic review is to provide a synthesis of educational and professional development needs and strategies for health-care providers in perinatal depression.

**Methods:**

A systematic search of the literature was conducted in seven academic health databases using selected keywords. The search was limited to primary studies and reviews published in English between January 2006 and May/June 2015, with a focus on perinatal depression education and professional development for health-care providers. Studies were screened for inclusion by two reviewers and tie-broken by a third. Studies that met inclusion criteria were quality appraised and data extracted. Results from the studies are reported through narrative synthesis.

**Results:**

Two thousand one hundred five studies were returned from the search, with 1790 remaining after duplicate removal. Ultimately, 12 studies of moderate and weak quality met inclusion criteria. The studies encompassed quantitative (*n* = 11) and qualitative (*n* = 1) designs, none of which were reviews, and addressed educational needs identified by health-care providers (*n* = 5) and strategies for professional development in perinatal mental health (*n* = 7). Consistently, providers identified a lack of formal education in perinatal mental health and the need for further professional development. Although the professional development interventions were diverse, the majority focused on promoting identification of perinatal depression and demonstrated modest effectiveness in improving various outcomes.

**Conclusions:**

This systematic review reveals a lack of strong research in multi-disciplinary, sector, site, and modal approaches to education and professional development for providers to identify and care for women at risk for, or experiencing, depression. To ensure optimal health outcomes, further research comparing diverse educational and professional development approaches is needed to identify the most effective strategies and consistently meet the needs of health-care providers.

**Trial registration:**

A protocol for this systematic review was registered on PROSPERO (Protocol number: CRD42015023701), June 21, 2015.

**Electronic supplementary material:**

The online version of this article (doi:10.1186/s12884-017-1431-4) contains supplementary material, which is available to authorized users.

## Background

According to the World Health Organization, the development of new knowledge and technologies requires deliberate efforts to ensure that clinical practice keeps pace with innovations [1]. Opportunities for ongoing, continuing education and professional development for health-care providers are essential to refine competencies and promote safety and quality care. In the area of perinatal care, nurses, midwives, and all health-care providers must especially demonstrate evidence-informed knowledge, skills, attitude, and judgement when considering conditions that can present with serious consequences for women and their babies. Specifically, perinatal depression is a mental illness that is often stigmatized, under-recognized, and undertreated as a mood disorder, despite its associated serious negative, and potentially life-threatening, outcomes [2, 3]. Perinatal depression is a specific depression that can occur during pregnancy and/or up to one year postpartum and affects nearly 13% of women [4]. Early recognition is particularly difficult given that it is often an invisible mental illness and difficult for health-care providers to detect. Many symptoms of perinatal depression have the potential to overlap with those of pregnancy, particularly somatic symptoms such as sleep disturbances and irritability, weight gain or loss, making accurate detection and treatment challenging. In addition, feelings experienced during infant care-taking such as self-doubt or worrying can also resemble perinatal depression [3, 5, 6]. Therefore, it is critical for health-care providers to accurately differentiate the various signs and symptoms of perinatal depressive disorder to ensure appropriate care [3, 5, 6].

A lack of concrete, continuing education and professional development strategies being implemented consistently and strategically can create barriers to nurses, midwives, and all health-care providers possessing the necessary skills and competencies to effectively detect symptoms and deliver high-quality, evidence-based care to perinatal women experiencing depression. Lack of education may also contribute to stigmatization and negative attitudes expressed by health-care providers, further jeopardizing women’s care [7, 8]. In this paper, continuing education and professional development is described as purposeful and often structured learning, occurring subsequent to entry-to-practice, with the goal of knowledge development and practice refinement for enhanced health services. It is widely recognized that the responsibility for continuing education is shared among individual health-care providers, groups of providers, and their workplace. Regardless of whether continuing education and professional development is mandatory, voluntary, formal or informal, the positive outcomes are many and range from personal satisfaction to professional competency which optimizes safe contemporary evidence-informed practice and quality health services for individuals and their families. In turn, continuing education and professional development mitigates outdated practice and protects the public [9].

Therefore, to address the challenge of perinatal depression detection and care in practice, continuing education and professional development strategies require a harmonized effort of clinicians, educators, employers, researchers and decision makers to contribute to the ongoing advancement of knowledge. For educators, in clinical and academic settings, Krugman and Warren [10] suggest that professional development requires a strategic approach to advance the professional and the profession as a whole. In addition, education offers a means for health-care providers to assess their own attitudes and stigmatization towards women with perinatal depression, in order to take steps to address negative beliefs [11]. Thereby, the authors of this study present a systematic review of the literature that critically examines the evidence on educational and professional development needs and strategies for health-care providers that are focused on prevention, detection, assessment, and care for perinatal depression.

## Methods

This systematic review used and adhered to the PRISMA guidelines for reporting systematic reviews [12]. It is part of a larger initiative to develop a best practice guideline at the Registered Nurses’ Association of Ontario (RNAO) entitled *Assessment and Interventions for Perinatal Depression, Second Edition* [13]. This guideline was developed to update the former 2005 guideline [14] and was expanded to include altered mood during both pregnancy and postpartum. More information on how to access the complete, updated best practice guideline can be found in Additional file 1.

The research question used to guide this review was: What education and professional development is required by health-care providers to ensure effective screening, assessment, and interventions for perinatal depression? The research question was developed and modified by the research team at the RNAO (consisting of two Master’s prepared nursing research associates [NRA] and a Master’s prepared program manager [PM]) and two PhD prepared co-chairs of the external guideline panel of experts. The research question was further adjusted and finalized based on input from the entire guideline development expert panel.

### Search methods and inclusion/exclusion criteria

A comprehensive search strategy was developed and run in seven academic health databases between May and June 2015 (the first search was run on May 20, 2015), with the assistance of a Health Sciences Librarian. The databases searched included CINAHL, MEDLINE, MEDLINE In Process, Cochrane Library (Cochrane database of Systematic Reviews and Cochrane Central Registry of Controlled Trials), EMBASE, ERIC, and PsycINFO. A protocol for this systematic review was also registered on the PROSPERO website (Protocol number: CRD42015023701). Studies had to be relevant to assessing knowledge/experience, or providing education and/or professional development to health-care providers or students on antenatal, perinatal, or postpartum depression in pregnant women or women less than one year postpartum. The search strategy therefore included MEDLINE MeSH headings and keywords that were relevant to perinatal (antenatal and/or postpartum) depression (“depression, postpartum” OR “puerperal disorders” OR postpartum depress* OR postnatal depress* OR antenatal depress*) and educational interventions (“education” OR “education, professional” OR “education, continuing” OR “education, professional, retraining” OR “education, distance” OR “education department, hospital” OR educat* OR train* OR health knowledge, attitudes, practice), specifically for nurses and other health-care providers (“health personnel” OR “personnel, hospital” OR “nurses” OR “health occupations” OR nurs*). The three search strings were combined using the Boolean operator AND. Comparable search terms were used when searching all seven databases and the full search strategy, including all search terms and applied limits, can be found in Additional file 1.

The inclusion criteria were any primary quantitative or qualitative research studies, mixed methods, systematic reviews, meta-analyses or meta-syntheses studies that were published between January 2006 and the day the search was run (a two-week period between May 20 and June 1, 2015). Articles were included if the content being studied (e.g., screening) was within a nursing or midwifery scope of practice and related to perinatal mental health. Articles were also included if they assessed existing experience or knowledge of perinatal mental health in health-care providers whose scope of practice overlaps with nursing or midwifery. Studies were excluded if they were not published in English, if they were consensus documents, discussion papers, case studies, case series, or if they were unpublished (e.g., grey literature).

### Screening process

The two NRAs and PM screened the title and abstracts against the systematic review inclusion criteria. The NRAs and PM divided the database of titles and abstracts into three groups and worked in pairs to flag studies that should move to the next stage of review. All screening was performed independently by at least two reviewers. Any discrepancies between each pairs’ results were tie-broken by the individual who did not review that group of articles. The full-text of articles that were included from the title and abstract screening stage were then reviewed for relevance. The NRAs and the PM again divided the articles into three groups and at least two reviewers independently reviewed articles for inclusion. Discrepancies from this stage were once again tie-broken by the individual who did not review that group of articles. The studies that passed the relevance review stage were then quality appraised and data extracted.

### Quality appraisal and data extraction

The quality of articles was assessed using the Critical Appraisal Skills Program (CASP) tools for the various study designs [15]. Quality appraisal was performed by the two NRAs and they appraised articles as weak (<62.5%), moderate (62.5% to 82.4%), or strong (>82.5%) depending on the CASP scores and using RNAO’s scoring system. To ensure reliability between the two reviewers during the appraisal process, a Kappa score was calculated. The NRAs independently quality appraised 20% of the total yield of articles that reached data extraction for all four systematic reviews used to inform guideline development, which was 26 of 130 articles (see Additional file 1 for access to full description of guideline development process). The NRAs reached a Kappa Score of 0.88, indicating high agreement (a score greater than 0.61 is considered acceptable agreement), and all remaining articles were then divided amongst the two reviewers for independent appraisal. No articles were excluded from this systematic review based on the quality appraisal scores.

Independent data extraction was performed by both the NRAs and the PM. A standardized data extraction table was used for each study and included information on the study’s quality score, level of evidence, objective, design, methods, sample, setting, analysis, outcome measures, results, conclusions, and limitations. Data extraction was performed by reviewing the full text article, removing the key elements, and populating the data extraction table in a Microsoft word document. All data extraction tables were kept in one master file prior to data synthesis.

### Data synthesis

Narrative summaries were used to group categories of similar articles together once the data extraction tables were complete. The summaries were also created by the NRAs and PM in an iterative and collaborative process. Articles were first sorted into categories based on the focus of the educational intervention or targeted education need and a narrative summary of the research for that category was created. This was not a meta-synthesis, but rather a process to delineate similar findings in a way to enhance clarity of large amounts of data. The narrative summaries included the name of the category in which articles were grouped, the strength and generalizability of the articles, the conclusions or conflicting results, and any major limitations present within the studies. The expert panel also used these summaries to help in the formation of recommendations for the guideline.

## Results

### Study selection

**Fig. 1:**
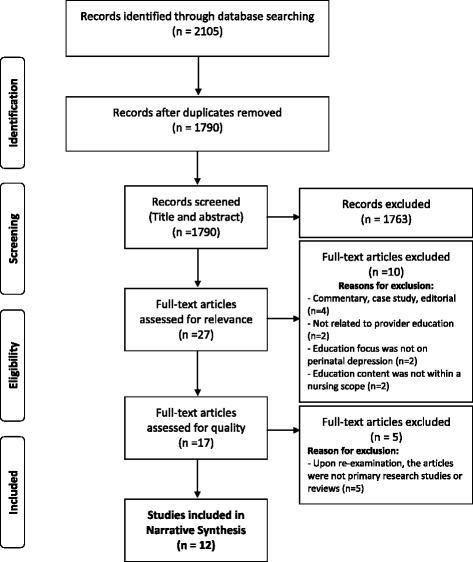
PRISMA flow diagram showing the inclusion and exclusion of articles during the systematic review process

The total number of articles returned from the database search after applying methodological limits was 2105. After removing duplicates, the total number of articles for title and abstract screening was 1790. Of these, 27 articles met inclusion criteria of having a focus on perinatal depression and health-care provider education and were taken to the relevance review stage. After relevance review, 17 articles were included in the quality appraisal process. Upon re-examination, an additional five articles were removed prior to data extraction. Although these five articles described the use of educational programs, they did not meet our inclusion criteria of being a primary research study or review and were thus excluded. This left 12 articles with a focus on perinatal mental health and continuing education and professional development in practicing health-care providers that were quality appraised, data extracted, and categorized into narrative summaries used to inform the findings for this systematic review. A detailed PRISMA flow diagram was created to track article inclusion and exclusion at all stages of the systematic review (Fig. 1) [12].

### Summary of study characteristics

The characteristics of the 12 included studies are provided in Table 1. All studies were published between 2006 and 2014 in Australia [7, 16–19], the United Kingdom (UK) [20–22], Scotland [8, 23], France [24], and Iceland [25]. The majority of the studies were conducted or involved providers in acute care settings (*n* = 9) [7, 8, 16, 17, 19–22, 24], two involved a range of provider setting [18, 23], and only one study occurred in a community health centre [25]. There were various study designs represented in the 12 articles, with the majority being quantitative (*n* = 11). The designs included quasi-experimental [16, 19, 20, 23], descriptive, cross-sectional studies [7, 17, 18, 21], a before-and-after design [19], a controlled trial [24], a randomized controlled trial [22], and one qualitative study [8]. No reviews or mixed methods studies were found. A total of 2818 provider participants were represented in the 12 studies, with sample sizes ranging from 21 to 1153 (with two studies having unspecified provider samples [22, 25]). The types of providers that the studies sampled included midwives [7, 16–20, 23, 24], nurses [8, 17, 21, 25], physicians (or general practitioners) [16, 17, 21], and/or health visitors [22]. Health visitors were the focus of a study conducted in the UK, and their role is generally defined as “registered nurses/midwives who have additional training in community public health nursing” [26]. Overall, the literature was divided into two distinct bodies: educational and professional development needs of health-care providers on perinatal depression, and professional development strategies for health-care providers on perinatal depression.

**Table 1:** Summary of Study Characteristics

Category	Citation	Study Design	Setting (Country)	Target Audience	Educational Intervention or Knowledge Assessment	Key Findings	Quality Rating^a^
Identifying perinatal mental health continuing education needs	Jones, Creedy, and Gamble [18]	Descriptive – cross-sectional	Australia	Practicing, student and non-practicing midwives (*n* = 815).	A survey was distributed to midwives and collected demographic information and previous educational preparation for the screening and care of women with antenatal and/or postpartum depression, as well as how adequate they perceived this training. 20 multiple choice questions also assessed knowledge of antenatal/postpartum depression.	The sample received an average score of 62.9% on antenatal depression and 70.7% on postpartum depression. A minimum score of 75% deemed a topic to be known. Many midwives felt their educational preparation was not adequate.	M
	Buist et al. [17]	Descriptive – cross-sectional	Australia	General practitioners (GP), maternal child health nurses (MCHN) and midwives (*n* = 1153).	Participants answered questions in a hypothetical vignette and a knowledge questionnaire about perinatal women. The hypothetical vignette required providers to consider what a women’s perinatal concern was and what would be the best course of action to take.	MCHN had higher levels of awareness of perinatal depression. GP and MCHN were more likely than midwives to recognize the need for providing help to women in emotional distress.	M
	McCauley, Elsom, Muir-Cochrane, and Lyneham [7]	Exploratory descriptive study	Australia	Midwives from 20 hospitals (*n* = 161).	A questionnaire was modified from two existing instruments that were used to survey mental health workers’ skills and knowledge. The questionnaire was distributed to midwives to explore their attitudes, skills, knowledge and experiences of working with perinatal women who have a mental illness.	Few participants in this study reported receiving any training in mental health and in many cases, mental health skills and knowledge were not seen by midwives as important in their work.	M
	Hardy [21]	Descriptive study	United Kingdom	Nurses (*n* = 390), GPs (*n* = 14), and clinical commission group (CCG) leads (*n* = 14).	Questionnaires were distributed to nurses, GPs and CGCs leads. The questionnaires for nurses had questions related to demographic information and perceived responsibilities and training needs regarding mental health and wellbeing. GPs and CGCs were asked fewer questions.	Nearly half of the nurses (42%) reported having had no training in mental health and wellbeing. 60.1% of nurses, 78.6% of CCGs and 53.8% of GPs felt that nurse also require training in managing mild to moderate depression and anxiety.	M
	McConachie and Whitford [8]	Qualitative, exploratory study	Scotland	Mental health nurses (*n* = 19).	Focus groups were conducted with mental health nurses to understand insights and attitudes towards the care of women with a severe mental illness during the perinatal period. Their experience working with perinatal women was explored through analyzing transcribed focus group interviews.	Four themes were identified: 1) normalization, 2) fear and anxiety, 3) frustration, and 4) confidence. Generally, mental health nurses had little experience and felt uneasy working with perinatal women.	M
Educational and professional development strategies to advance knowledge and skills in perinatal mental health	Jardri et al. [24]	Controlled trial	Lille, France	Midwives (*n* = 21).	A three-hour perinatal depression assessment training course for midwives was offered. The course had five sections: 1) general data about perinatal depression, 2) risk markers for perinatal depression, 3) examination of previous screening, 4) recommendations from the department, and 5) how to use the EPDS appropriately. A summary of recommendations were sent to each midwife following the course.	Correlation between midwife assessments and diagnosis of perinatal depression improved following training (significantly). The training course led to a 37.7% improvement in diagnosis of major depressive episodes.	M
	Morrell et al. [22]	Cluster randomized trial	Trent, England	Health visitors in 101 general practices.	A psychologically-informed approach was used to train health visitors on identifying perinatal depressive symptoms using the EPDS and assess mood and suicidal thought. The participants were trained to deliver psychologically-informed sessions based on *either* cognitive behavioural principles or person-centred principles.	At six months, 34% of women in the intervention group (had providers who received training) and 46% of women in the control group had a score ≥ 12, indicating a reduction in symptoms in the intervention group.	M
	Ingadottir and Thome [25]	Controlled quasi-experimental intervention study	Reykjavik, Iceland	Nurses in 6 community health centres.	A web-based course focusing on evidence-based interventions for postpartum emotional distress was offered to nurses. There were seven self-study units which focused on postpartum emotional distress and nursing interventions, interventions for stress and fatigue, irritable infants, and a case study on a postpartum emotional distress and the effect on the spouse.	At 15 and 24 weeks there was a significant difference between mothers’ EPDS scores at the experimental and control centres in favour of the intervention group of nurses who received the web-based course.	M
	Hegarty et al. [16]	Quasi-experimental, pre-post test	Australia	Staff on a maternity unit (*n* = 27) (21 midwives, 5 doctors, 1 multi-cultural worker).	Staff were trained using a new (ANEW) program based on a previous postnatal program, conducted over 26 weeks with a commitment of 1–2 h per week. The goal of ANEW was to enhance the knowledge and skills of providers to identify and support women experiencing psychosocial issues during pregnancy. Evidence-based resources were provided and there were four interactive workshops using role play and feedback with simulated patients.	There was a significant increase in the proportion of women who reported that midwives helped them to talk about emotional and social issues in the post-intervention group (ANEW).	W
	King, Pestell, Farrar, North, and Brunt [20]	Quasi-experimental, pre-post test	United Kingdom	Hospital maternity staff (*n* = 126), community maternity staff (*n* = 23).	Community hospital maternity staff took part in attending psychological assessment training. The course took place over three, 2-h sessions. The training was conducted by a clinical psychologist or a counsellor. Staff were asked to rate their levels of confidence before and after the training.	T-tests showed that staff confidence in all areas was significantly increased by attending the training program.	W
	Forrest [23]	Quasi-experimental, post-test only	Scotland	Midwives and their mentors (*n* = 30).	The Perinatal Mental Health Curricular Framework was developed to address the educational needs of providers. A multi-disciplinary approach guided the educational material, and it was applied using both e-learning and face-to-face learning. Two, day-long workshops were held and modules were introduced online. Assessment was performed using a modified online examination. The professional role of the module facilitator was not specified.	All students successfully passed the exam. Collectively, the feedback was mainly positive. The multidisciplinary aspect was highly favoured by all students and they welcomed gaining insight into other professional approaches to perinatal mental health.	W
	McLachlan, Forster, Collins, Gunn, and Hegarty [19]	Before-and-after survey design	Victoria, Australia	Midwives at two hospitals (*n* = 25).	An advanced communication skills education package was delivered. Midwives attended seven sessions over a six-month period for a four week course, with three two-hour workshops facilitated by a psychologist. Workshop topics explored the aspects of providing woman-centred care for perinatal women. The program used evidence-based resources as well as role play and feedback with simulated patients.	Midwives felt more confident in their knowledge of psychosocial issues and in supporting women experiencing these issues in the early postnatal period following the educational program.	W

^a^Quality rating scores are abbreviated. ‘W’ reflects a weak rating and ‘M’ reflects a moderate rating. There were no studies rated strong

The quality appraisal identified that four of the 12 studies were rated as weak in methodological quality [16, 19, 20, 23] and eight were rated as moderate in methodological quality [7, 8, 17, 18, 21, 22, 24, 25]. No studies were appraised as strong methodological quality. The lack of strongly rated literature was largely due to sources of bias within the various study designs. Sources of bias within the cross sectional, descriptive studies included low response rates, a lack of externally validated and reliable measures, and a limited description of data analysis and the exact methods of data collection. The quasi-experimental also had sources of bias. These studies commonly did not blind participants or researchers, did not include a power calculation for determining sample size, and had high participant loss to follow-up. The remaining study designs had many of the same issues. The methodological rating of all included studies can be found in Table 1.

### Professional development and education literature

As previously stated, the 12 studies were divided into two distinct categories; 1) identifying perinatal mental health continuing education needs (*n* = 5), and 2) educational and professional development strategies to advance knowledge and skills in perinatal mental health (*n* = 7). The category of identifying perinatal mental health educational needs examined studies that assessed providers’ level of knowledge and experience in perinatal depression. As a result, these studies also provided insight into existing gaps in perinatal mental health education and professional development for health-care providers. The category of educational and professional development strategies were studies that provided opportunities to promote providers’ assessment and intervention practices for pregnant and postpartum mothers and was further subdivided into those strategies that focused on only the antenatal period and those that focused more broadly on the perinatal period. The antenatal period referred to the time before the mother gives birth (pregnancy) and the postpartum period referred to time following birth, up to one year. The perinatal period refers to the time between early pregnancy *and* up to one year following birth.

### Identifying perinatal mental health continuing education needs

There were five studies that addressed existing gaps in health-care provider education and professional development needs that must be addressed to advance practice in perinatal mental health. Two of these studies focused on identifying the knowledge, perceptions, and needs of midwives working with perinatal women [7, 18]; two of the studies focused on identifying knowledge and needs of multidisciplinary providers including general practitioners (GPs), nurses, midwives, and clinical commissioning group (CCG) leads [17, 21]; and one study focused on exploring the experiences and attitudes of mental health nurses caring for perinatal women with mental illness [8]. The approaches for identifying needs and assessing knowledge and attitudes varied between the studies, including the use of both objective and subjective measures. Two studies used surveys with multiple choice questions or hypothetical vignettes to assess health-care providers’ knowledge levels [17, 18]; two studies used questionnaires to assess perceived level of knowledge, attitudes, or training needs [7, 21]; and one study used a focus group to assess experiences and attitudes [8]. Despite the variation in methods and measures, the majority of the literature indicated that there was little education and training in regards to perinatal mental health, which often negatively impacted their levels of knowledge and awareness on these issues, particularly in midwives.

Knowledge questionnaires were used in two cross-sectional studies to assess the level of awareness possessed by midwives [18], and GPs, maternal child health nurses, and midwives [17], in regards to perinatal mental health issues. The knowledge questionnaire distributed to only midwives (*n* = 815) in a national Australian study also collected information on educational preparation for the screening and care of women with perinatal depression [18] and found that on average most midwives felt that their educational preparation was not adequate, with little focus on antenatal or postpartum depression. On average, the sample scored 62.9% of the questions on antenatal depression and 70% of the questions on postpartum depression correctly, indicating a reasonable, but not high-level of knowledge on the topics [18]. The respondents in another Australian cross-sectional study scored slightly better when answering questions using a hypothetical vignette and a knowledge questionnaire [17]. Overall, maternal child health nurses were found to have higher levels of awareness of perinatal depression than GPs or midwives [17]. GPs and maternal child health nurses were also more likely than midwives to recognize the need to provide help for women with emotional distress, indicating a potential need for further midwifery education in this area [17]. In all providers, neither level of knowledge nor years of experience significantly affected the responses to the vignette [17].

Two studies also examined providers’ perceived level of knowledge and skills through questionnaires distributed to midwives [7], and nurses, GPs, and CCG leads [21]. The questionnaire distributed to 161 midwives in an Australian descriptive study found that few respondents reported receiving any training in mental health as it relates to perinatal women. Furthermore, mental health skills and knowledge were also not consistently seen by the surveyed midwives as important in their work [7]. This study highlighted the need for not only further education and training in perinatal mental health for midwives, but also the need for support from other mental health professionals to act as a resource for consultation and referral as needed [7]. The questionnaire distributed to nurses (*n* = 390), GPs (*n* = 14) and CCG leads (*n* = 14) in another descriptive study conducted in the UK found similar results, specifically in regards to the lack of previous nursing education in mental health and wellbeing [21]. Nurses most often indicated their preference for face-to-face training in a classroom environment and over 50% of the nursing respondents felt that they needed training regarding maternal mental health [21]. Similar percentages of the GP (53.8%) and CCG (78.6%) respondents also felt that nurses needed this kind of tailored professional development and education on maternal mental health issues, as well as for intervening for mild to moderate depression and anxiety [21].

A qualitative exploratory study conducted in Scotland employed the use of focus groups with mental health nurses (*n* = 19) to explore their experiences and attitudes of providing care to women with a severe mental illness during the perinatal period [8]. From this focus group, four themes were identified which included normalization, fear and anxiety, frustration, and confidence. Many participants normalized the mental illness by perceiving that perinatal mental health issues are no different than any other mental health issues [8]. Participants also expressed a lack of confidence when providing care to women in the perinatal period experiencing mental health issues due to a lack of experience within this area [8]. They also expressed their frustrations in regards to the stigmatization that can come from screening and mental health referrals for women in the perinatal period [8]. Finally, most participants highlighted some good experiences of multi-professional teamwork when working with women in the perinatal period experiencing mental illness, which was seen as both reassuring and enhancing confidence [8].

### Educational and professional development strategies to advance knowledge and skills in perinatal mental health

Strategies for advancing education and professional development in health-care providers working with populations at risk for perinatal mental health concerns were addressed within seven studies. Specifically, five out of seven of these intervention studies were focused on the professional development and education of midwives [16, 19, 20, 23, 24], with the remaining studies considering the education of registered nurses [25], physicians and midwives [16], and health visitors [22]. The focus of most studies was providing education to those providers who work specifically within an acute care setting, with only one study considering the needs of those working in community health centres [25]. The educational content in this category is further divided into two subcategories: 1) those that focus on identifying and providing care for depressive symptoms during the antenatal period [16, 20]; and 2) those that focus on identifying and providing care for depressive symptoms during the perinatal period [19, 22–25].

#### Antenatal educational and professional development strategies

Two papers presented professional development strategies that primarily focused on educational content for identifying antenatal depressive symptoms [16, 20]. An Australian program called a new (ANEW) approach was developed for health-care provider professional development on identifying psychosocial issues and communicating with women about these issues [16]. It consisted of supplying midwives (*n* = 21) and physicians (*n* = 5) with evidence-based resources, four interactive group workshops, and six sessions every two weeks to discuss the participants’ experiences over 26 weeks [16]. There was a one-to-two hour commitment required from provider participants each week. The program was evaluated by two groups of pregnant women; one group of women completed surveys before the provider intervention (*n* = 584) and a different group of women completed surveys after the provider intervention (*n* = 481) [16]. The researchers found that there was no statistically significant difference between the two groups of pregnant women for either their Edinburgh Postnatal Depression Scale (EPDS) scores or the communication skills of providers [16]. However, following the ANEW program, there was a statistically significant increase in the proportion of women who would discuss worries related to coping after child birth, specifically with midwives (pre-ANEW: 77.1%, post-ANEW: 83.6% [OR 1.51, CI 1.10–2.08]) [16]. There was also an increase in the proportion of women who reported that midwives specifically assisted them in discussing emotional and social issues following ANEW (pre-ANEW: 20.3%, post-ANEW: 27.2% [OR 1.47, CI 1.09–1.98]) [16].

Similarly, a study based in the UK focused on providing psychological assessment skills education to community midwives and maternity staff (*n* = 23) and maternity hospital staff (*n* = 126) related to screening and monitoring of pregnant women during the antenatal period [20]. The course included three, two-hour sessions provided in person by a clinical psychologist or counsellor and provided education on several areas of assessment and monitoring, as well as the use of specific screening tools [20]. The staff reported improvement in several areas of confidence and all areas showed a significant improvement post-education. However, an identified concern from participants was difficulty finding time to complete and score the screening tool [20]. The commonality between these two studies was their in-person, interactive teaching approach provided over more than one session, which seemed to yield positive responses.

#### Perinatal educational and professional development strategies

The remaining five studies focused on educational content to identify depressive symptoms in the perinatal period and providing care to women experiencing these symptoms [19, 22–25]. One quasi-experimental study from Scotland utilized a multiprofessional educational approach to guide the educational material and content for midwives and their mentors (*n* = 30) on prevention, detection, and management of mental health and other perinatal outcomes [23]. This approach was used to teach the midwives aspects of other professionals’ approaches to perinatal mental health and was applied using blended learning to ensure flexibility, which encompassed face-to-face and online components, as well as required workshops and modules [23]. The multiprofessional aspect of learning other professional approaches to perinatal mental health was highly favoured by all participants, and the overall feedback of the education program was positive [23].

Two studies tailored the majority of their educational content specifically on identifying women experiencing perinatal depression and used outcome measures related to the detected rates of depression. A controlled trial conducted in France used a one-time, three-hour training course to teach new recommendations for screening to midwives (*n* = 21) [24]. The training included content on risk markers for perinatal depression and how to properly administer the EPDS, with content summaries provided following the training [24]. The training was evaluated based on midwives’ effectiveness of screening a group of postpartum women pre- and post-training and their screening was assessed using a questionnaire designed for the study (MidQ). EPDS scores completed by postpartum women were compared to the MidQ scores to determine effectiveness in detecting depression pre- and post-training. When comparing EPDS scores to the midwives MidQ results, screening by midwives improved significantly following training, demonstrating a 37.7% (95% CI 25.7–49.7) improvement in the detection of major depressive episodes [24].

A cluster randomized trial took a similar approach and taught health visitors, from 101 general practices in the UK, content on screening and providing follow-up sessions to mothers using psychologically informed approaches [22]. The psychologically informed approaches were based on principles of cognitive behavioural therapy or person-centred principles and were taught for one hour a week for eight weeks. This approach also used monthly reflective practice training sessions as a way to keep health visitors up-to-date on their training [22]. Upon completion of the provider intervention, postnatal women with EPDS scores greater than 12 were randomized into either an intervention (*n* = 2249) or control group (*n* = 1335) to evaluate the effectiveness of training on women’s outcomes [22]. The intervention group received care from providers who had received the training and the control group received usual provider care. The results demonstrated a positive effect in reducing the proportion of women with depressive symptoms at six months postpartum (12.4% of women in the intervention group had EPDS scores greater than 12, compared to 16.7% in the control group) [22]. The results were also maintained at 12 months postpartum [22].

An online course was also implemented for nurses from three community health centres in Iceland using a self-study approach to teach content on identifying and caring for emotional distress and depression in women postpartum [25]. In this quasi-experimental study, nurses were required to read each unit and post discussions to an online forum [25]. The content also stressed the importance of providing home visits between 9 and 15 weeks to mothers scoring greater than 12 on the EPDS. A control group of nurses from another three community health centres did not receive the intervention. The intervention group had significantly more contact with new mothers suffering from emotional distress, and used more evidence-based approaches compared to nurses providing usual care [25]. There were also found to be significantly lower EPDS scores for mothers at 15 and 24 weeks postpartum at the community health centres implementing the professional development strategy (*p* > 0.05) [25].

Another approach for delivery of professional development education to midwives working with postpartum women was implemented using a before-and-after study design [19]. This Australian study employed the use of an educational package designed to advance communication skills for midwives (*n* = 25) caring for postpartum women and involved a commitment of two hours per week for four weeks [19]. The program used evidence-based resources, role-play and feedback with simulated postpartum women to enhance learning [19]. There were also three additional two-hour workshops facilitated by a psychologist. Following the educational intervention, midwives were more likely to report competence in engaging in dialogue with women on psychosocial issues and emotional health (*p* = 0.02) [19]. They also reported more confidence in their knowledge of psychosocial issues (*p* = 0.01) and their ability to support postpartum women experiencing similar issues (*p* = 0.02) [19]. However, this study did not measure any outcomes for women following the delivery of the professional development education intervention.

## Discussion

### Summary of findings

The purpose of this systematic review was to synthesize the evidence regarding educational and professional development needs and strategies for health-care providers in perinatal depression. Our review only found 12 articles of weak to moderate quality, none of which were systematic reviews. The studies that examined educational and professional development needs of health-care providers found a paucity of previously provided education and professional development in regards to perinatal mental health, which often negatively impacted levels of knowledge and awareness on these issues, particularly in midwives. Nevertheless, the findings overall suggest that diverse professional development strategies on identifying and caring for women with perinatal depression have been recognized as enhancing various outcomes for health-care providers including nurses [25], midwives [16, 19, 23, 24], physicians [16], and/or health visitors [22]. Intervention studies demonstrated improved practitioner confidence [19, 20, 23], increased levels of knowledge [23], improved screening efficiency [24], and overall favourable outcomes for women (e.g., reduced EPDS scores, improved communication) [16, 22, 25] without any reported harms or adverse events.

Positive outcomes occurred regardless of whether the content was focused on assessment and care during the antenatal, perinatal, or postnatal period, and regardless of diverse modalities used (e.g. face-to-face teaching, web-based programs, and case-based vignettes). This suggests that regardless of how the content is delivered or for how long, any professional development education on perinatal depression will enhance some aspects of provider confidence or knowledge and is certainly more effective than no education at all. However, it is important to note that although all intervention studies had some beneficial effects, suggesting the appropriateness of several professional development modalities, none of the studies specifically compared different approaches to evaluate outcomes. For this reason, we cannot draw definitive conclusions regarding which strategies would be considered the most effective in practice for detection, assessment, or interventions for perinatal depression.

### Research implications

Overall, there was limited recent, strong-quality research available that examined educational and professional development strategies for health-care providers on perinatal depression. However, based on the existing findings and our analysis of the evidence, we provide four recommendations for future research. First, although it has become clear from the existing research that we are not yet aware of the most effective strategy for professional development in perinatal depression, it is even less apparent what content and strategies are necessary to integrate into undergraduate education for health-care providers. None of the included studies specifically assessed strategies administered in undergraduate curriculum, yet many of the included studies addressed the lack of previous education in the area of perinatal mental health [7, 18, 21]. Continuing education and professional development will always be an ongoing endeavour, but providing health-care providers with comprehensive content on perinatal depression prior to beginning practice would contribute to an increased foundation of knowledge in this area. Future research should consider exploring strategies that use theoretical concepts and opportunities for clinical practice on perinatal depression in undergraduate curriculum, integrating the role of nurses, midwives, and other health-care providers [7, 17, 27].

Second, research is needed to determine *how* to best deliver professional development to health-care providers that aligns with their professional standards and *who* is best suited to provide this type of ongoing education. The approaches suggested within our results can be used as a reference point for educators, with the understanding that this review cannot definitively suggest effectiveness of all interventions and who is best suited to design and implement the approaches. It is critical that the educator of the material is not only experienced in the content, but also understands the various modalities to best deliver professional development strategies within their setting. Existing findings from our review spanned across many different geographic locations and professions, which has implications for the educational content and how it meets specific providers’ professional standards and scope of practice. In addition, as all of the studies were conducted in either Europe or Australia, future research studies should be adapted to explore continuing education in multiple geographic health-care contexts, including Asia, North America, and South America. Future research should also specify how the study’s professional development approach aligns with their region’s professional standards and specific providers’ scope of practice.

Third, future research should also incorporate elements of reflective practice into educational and professional development strategies and measure changes in outcomes of health-care providers’ attitudes and behaviours on perinatal depression. Very few of the included studies specifically described incorporating elements of reflective practice into the educational content [19, 20]. Although participants may have demonstrated increased knowledge and skills in perinatal depression following the interventions, negative attitudes may persist in the absence of reflective practice, resulting in potential barriers to care for perinatal women and less than optimal outcomes [7, 8, 19]. Future research should measure outcomes of attitudes and behaviours before and after reflective practice is incorporated into an educational strategy in order to determine effectiveness beyond what is captured from the perinatal content itself.

Fourth, more recent research is needed to support educational approaches that provide content on assessment and care of women with depression during the entire perinatal period. The included studies in our findings varied between tailored approaches for antenatal depression [16, 20], postpartum depression [25], or perinatal depression [19, 22–24]. The most recent literature stresses the importance of identifying symptoms of depression not only up to one year following childbirth, but also during pregnancy [13]. Therefore, educational strategies must be broad enough to capture the importance of assessment and care during the entire perinatal period, and future research should evaluate such approaches in a standardized and measurable way.

### Policy implications

In addition to the need for further research, specific clinical, organizational, and system-level changes are needed to help transform the culture of obstetric care settings to optimize depression treatment for perinatal women [28]. These changes must include ongoing education for health-care providers. The absence of more funded research and educational and professional development opportunities in this area may represent the existing negative attitudes or stigmatization towards perinatal women with depressive symptoms that continues to create barriers to care and improved outcomes. Our work on the best practice guideline that was informed from this systematic review’s findings is an effort in itself to address the existing gap in professional development, education, and research on perinatal depression [13]. For educators in clinical and academic settings, the guideline can offer a resource to enhance health-care providers’ confidence, attitudes and knowledge about perinatal depression. Additionally, this manuscript serves as a resource to highlight the professional development approaches that have been empirically tested, although not extensively.

When considering professional development opportunities, many organizations may not be fully aware of the extent to which there is a gap in knowledge on perinatal depression, and health-care providers may have varying degrees of education. Conducting a needs assessment within a maternity or mental health unit, or for a specific professional group regardless of setting, may be an important step to identifying the existing gaps in knowledge and the desired areas for education improvement. Specifically, McCauley, Elsom, Muir-Cochrane, and Lyneham found that midwives expressed the need for assistance from multidisciplinary professionals, including those in maternal-child care and mental health services, so that appropriate consultation and referral can occur for perinatal women in need [7]. Incorporating multidisciplinary professional development and educational opportunities into a health-care setting could amalgamate training into fewer sessions and also encourage collaboration among diverse professionals. Departments should also consider incorporating an opportunity for all new staff to receive, at minimum, orientation education on perinatal depression to ensure consistent integration of standardized practices. Promoting practice and education changes should lead to a departmental policy that supports an ongoing commitment to perinatal mental health.

We recognize that despite the importance of education and professional development on perinatal depression, considerations of cost and cost effectiveness must be made to justify the means. The findings from our review did not report cost and barriers to funding for continuing education and professional development for health-care providers. Funding opportunities, available resources, and time commitment for professional development may vary across health sectors and settings, and the extent to which the program can be delivered (e.g., a three-hour workshop or a six-week course) must be taken into consideration by administrators and clinical educators. However, organizations should prioritize education and professional development where possible for members of the health-care team. Organizations should also consider designating those health-care providers who received formal education as champions or mentors to serve as a resource for other staff. This may ultimately serve as a cost-savings measure by promoting knowledge and resource sharing among the health-care team.

### Limitations

This systematic review was conducted using a rigorous methodology; however, it is not without limitations in both our methods and the methods of the included studies. There were only a total of 12 research studies that were able to inform our findings, none of which were rated strong in methodological quality. Many of the included studies had small sample sizes, used quasi- or non-experimental design, and were only conducted at one site, at one point in time. The approaches used to inform the educational programs of the included studies were also diverse, measured different outcomes, and targeted various groups of health-care providers. The results were also often based on self-reported scales, which brings into question the validity and reliability of the measures. Overall, these aspects limit the generalizability of our findings and the ability to make definitive conclusions on the effectiveness of any one educational or professional development approach. Furthermore, very few studies indicated who was providing the educational intervention (e.g., nurse, midwife, psychologist, etc.), making it difficult to indicate the ability for nurses, midwives, or other health-care providers to instruct the proposed material. The instructed material included in the studies was also often not described in explicit detail.

Although our search strategy was developed in accordance with a health sciences librarian, it is possible that relevant studies may have been missed. We also did not include studies published in languages other than English, did not include grey literature in our search, and did not search the reference lists of included studies for further evidence. Although we limited our search to peer reviewed literature, we did not contact study authors to obtain any missing data, which may introduce the potential for publication bias within this review. It is also important to note that as this was a nursing-focused initiative, we excluded studies that examined educational or professional development content beyond a nursing scope of practice. For this reason, articles focused on physician or resident continuing education or professional development strategies or content (e.g., training on appropriate anti-depressant medication dosing) would have been excluded.

## Conclusion

This systematic review is the first that we are aware of examining the continuing educational and professional development approaches for health-care providers working with women at risk for, or experiencing, perinatal depression. It has revealed a lack of research in multi-disciplinary, multi-sector (community and acute care), and multi-site (urban, rural, and remote) approaches to education and professional development that would ensure all health-care providers have the same understanding and complimentary approaches to identify women at risk for developing depression and to provide interventions to those experiencing depressive symptoms. The search also has not revealed a strong argument for one educational strategy or approach (e.g., e-learning strategies, in-person approaches) to guide those wanting to initiate programming for a particular professional or practice group.

There is a need for initial education and ongoing professional development to improve the knowledge of perinatal depression, identification and intervention skills of health-care professionals caring for perinatal women at risk. However, as this review has revealed, there is a dearth of strong published research on evidence-based educational strategies and approaches that will improve health-care providers’ knowledge and skills in perinatal depression prevention, detection, assessment, and interventions to ensure optimal health outcomes for women and their families. Further research and policy-based initiatives promoting professional development are needed in order to develop providers’ knowledge, competence, and confidence in perinatal depression and address the ongoing gap in educational needs.

## Additional file

Additional file 1: Full search strategy used for this systematic review, including all condensed search terms and the full inclusion and exclusion criteria. (PDF 375 kb)

## Abbreviations

CCG: Clinical commissioning group
EPDS: Edinburgh Postnatal Depression Scale
GP: General practitioner
MCHN: Maternal child health nurse
NRA: Nursing Research Associate
PM: Program Manager
RNAO: Registered Nurses’ Association of Ontario
UK: United Kingdom
