# Supplementary material for: Approaches to health-care provider education and professional development in perinatal depression: a systematic review
Source: BMC Pregnancy Childbirth. 2017 Jul 24;17:239. doi: 10.1186/s12884-017-1431-4 (PMC5525243; doi:10.1186/s12884-017-1431-4)
Supplement: Additional file 1: — Full search strategy used for this systematic review, including all condensed search terms and the full inclusion and exclusion criteria. (PDF 375 kb) [file 12884_2017_1431_MOESM1_ESM.pdf]

**Additional File 1.**
**Search Strategy**



**Systematic Database Search**
A systematic search for recent literature relevant to the scope of the research question was conducted by a health sciences librarian.

**List of Databases Searched**: Seven databases were searched in total: Cumulative Index to Nursing and Allied Health (CINAHL), Cochrane Library (Cochrane Database of Systematic Reviews and Cochrane Central Register of Controlled Trials), Embase, MEDLINE, MEDLINE In-Process, PsychINFO, and Educational Resource Information Centre (ERIC).

**Research Question:** What education and professional development is required by health-care providers to ensure effective screening, assessment, and management of perinatal depression?

**Inclusion Criteria:**
The following criteria were used to guide the search:

- A primary focus of the article is on the topic area: health-care provider education required for assessment and management of perinatal (antenatal or postpartum) depression in pregnant women or women with a newborn or infant less than 12 months. Education can focus on caring for women with co-existing mental health disorders during pregnancy or postpartum (e.g. anxiety) but primary focus of education must be on perinatal depression
- Study methodology limited to all types of primary studies: quantitative, qualitative, mixed methods, and reviews
- Published between January 2006 and June 2015
- Published in English
- Accessible for retrieval
- Applicable across health settings/sectors where nurses provide direct clinical care to pregnant or postpartum women
- Primary focus on nurses (registered nurses, registered practical nurses, nurse practitioners, etc.)
- Secondary focus on other health-care providers (whose scope of practice overlaps with nursing)
- Applicable to nurses in a Canadian context

**Exclusion Criteria:**
The following criteria were used to exclude literature from the search:

- Topic not related to education of health-care providers on perinatal depression, or focus of education is solely on other mental illnesses beyond the topic area (e.g., eating disorders, bipolar disorder, postpartum psychosis, postpartum blues, etc.).
- Dissertations, commentaries, narrative, anecdotal articles, letters to the editor, editorials, expert reports, consensus documents, discussion papers, case studies, case series
- Studies without specified methodology that cannot be quality appraised
- Non-English studies
- Unpublished (grey literature)
- Studies involving animals
- Articles focusing only on providing patient education related to perinatal depression
- Articles with a specific focus on providing education related to pharmacologic interventions

**Additional File 1.**
**Search Strategy**



**Search Terms**
Below are condensed search terms used for the condition and intervention in MEDLINE. Comparable terms were used in the additional databases searched.

| Patient Condition | | Provider Population | | Intervention | Limits Applied |
|---|---|---|---|---|---|
| OR | | OR | | OR | |
| 1-Depression, Postpartum/<br>2-Puerperal Disorders/<br>3-(postpartum adj2 depress*)<br>4-(post-partum adj2 depress*)<br>5-(postnatal adj2 depress*)<br>6-(post-natal adj2 depress*)<br>7-(antenatal adj2 depress*)<br>8-(ante-natal adj2 depress*)<br>9-(prenatal adj2 depress*)<br>10-(pre-natal adj2 depress*)<br>11-(antepartum adj2 depress*)<br>12-(ante-partum adj2 depress*)<br>13-(puerper* adj2 depress*)<br>14-(postpartum adj2 disorder?)<br>15-(post-partum adj2 disorder?)<br>16-(postnatal adj2 disorder?)<br>17-(post-natal adj2 disorder?)<br>18-(antenatal adj2 disorder?)<br>19-(ante-natal adj2 disorder?)<br>20-(prenatal adj2 disorder?)<br>21-(pre-natal adj2 disorder?)<br>22-(antepartum adj2 disorder?)<br>23-(ante-partum adj2 disorder?)<br>24-(puerper* adj2 disorder?)<br>25-or/1-24 | A<br>N<br>D | 26-Health Personnel/<br>27-Personnel, Hospital/<br>28-Health Occupations/<br>29-Professional Role/<br>30-Nurse's Role/<br>31-Licensed Practical Nurses/<br>32-exp Nurses/<br>33-exp Nursing Staff/<br>34-Nursing Staff, Hospital/<br>35-exp Nursing/<br>36-Nursing, Practical/<br>37-(health* adj2 provider?).mp,kw.<br>38-(health* adj2 staff?).mp,kw.<br>39-(health* adj2 personnel?).mp,kw.<br>40-(health* adj2 employe*).mp,kw.<br>41-(health* adj2 profession*).mp,kw.<br>42-(health* adj2 occupation?).mp,kw.<br>43-(health* adj2 practitioner?).mp,kw.<br>44-(profession* adj2 role?).mp,kw.<br>45-nurs*.mp,kw.<br>46-or/26-45 | A<br>N<br>D | 47-Education/<br>48-Education, Professional/<br>49-Education, Continuing/<br>50-Education, Nursing, Continuing/<br>51-Education, Professional, Retraining/<br>52-Education, Distance/<br>53-Education Department, Hospital/<br>54-Education, Public Health Professional/<br>55-exp Hospitals, Teaching/<br>56-Clinical Clerkship/<br>57-exp Professional Competence/<br>58-exp Education, Nursing/<br>59-exp Inservice Training/<br>60-Staff Development/<br>61-Preceptorship/<br>62-exp Teaching/<br>63-Teaching Materials/<br>64-exp Curriculum/<br>65-Videoconferencing/<br>66-Webcasts as Topic/<br>67-Mentors/<br>68-Learning/<br>69-Health Knowledge, Attitudes, Practice/<br>70-educat*.mp,kw.<br>71-((staff? or profession*) adj2 develop*).mp,kw.<br>72-(clinical adj2 clerkship?).mp,kw.<br>73-(professional* adj2 competen*).mp,kw.<br>74-train*.mp,kw.<br>75-preceptorship*.mp,kw.<br>76-preceptor-ship*.mp,kw.<br>77-teaching*.mp,kw.<br>78-curricul*.mp,kw.<br>79-(workshop? or work shop?).mp,kw.<br>80-seminar?.mp,kw.<br>81-orientation?.mp,kw.<br>82-session?.mp,kw.<br>83-round?.mp,kw.<br>84-webinar?.mp,kw.<br>85-videoconferenc*.mp,kw.<br>86-video-conferenc*.mp, kw.<br>87-audioconferenc*.mp,kw.<br>88-audio-conferenc*.mp, kw.<br>89-teleconferenc*.mp,kw.<br>90-tele-conferenc*.mp,kw. | 109-exp animals/ not (exp animals/ and exp humans/)<br>110-108 not 109<br>111-limit 110 to (case reports or comment or consensus development conference or consensus development conference, nih or duplicate publication or editorial or guideline or lectures or letter or news or newspaper article or practice guideline)<br>112-110 not 111<br>113-limit 112 to yr="2006 -Current"<br>114-limit 113 to english language |

| | | | | | |
|---|---|---|---|---|---|
| | | | | 91-conferenc*.mp,kw.<br>92-colloquium*.mp,kw.<br>93-webcast*.mp,kw.<br>94-web-cast*.mp,kw.<br>95-elearn*.mp,kw.<br>96-e-learn*.mp,kw.<br>97-mentor*.mp,kw.<br>98-coaching*.mp,kw.<br>99-learning*.mp,kw.<br>100-(knowledge adj3 attitude?).mp,kw.<br>101-awareness*.mp,kw.<br>102-skill?.mp,kw.<br>103-(theory or theories).mp,kw.<br>104-abilit*.mp,kw.<br>105-concept?.mp,kw.<br>106-stigma?.mp,kw.<br>107-or/47-106<br>108-25 and 46 and 107 | |

Access to the complete best practice guideline and full details on the guideline development process informed by four systematic reviews can be found at:
**http://RNAO.ca/bpg/guidelines/perinatal-depression**
